# Physical exercise in aging human skeletal muscle increases mitochondrial calcium uniporter expression levels and affects mitochondria dynamics

**DOI:** 10.14814/phy2.13005

**Published:** 2016-12-30

**Authors:** Sandra Zampieri, Cristina Mammucari, Vanina Romanello, Laura Barberi, Laura Pietrangelo, Aurora Fusella, Simone Mosole, Gaia Gherardi, Christian Höfer, Stefan Löfler, Nejc Sarabon, Jan Cvecka, Matthias Krenn, Ugo Carraro, Helmut Kern, Feliciano Protasi, Antonio Musarò, Marco Sandri, Rosario Rizzuto

**Affiliations:** ^1^ Ludwig Boltzmann Institute of Electrical Stimulation and Physical RehabilitationViennaAustria; ^2^ Venetian Institute of Molecular MedicinePadovaItaly; ^3^ Department of Biomedical ScienceUniversity of PadovaPadovaItaly; ^4^ DAHFMO‐Unit of Histology and Medical Embryology, IIMInstitute Pasteur Cenci‐BolognettiSapienza University of RomeRomeItaly; ^5^ Department of Neuroscience, Imaging and Clinical SciencesCeSI‐Met – Center for Research on Aging and Translational Medicine & DNICSUniversity G. d'AnnunzioChietiItaly; ^6^ Science and Research CentreInstitute for Kinesiology ResearchUniversity of PrimorskaKoperSlovenia; ^7^ Faculty of Physical Education and SportComenius UniversityBratislavaSlovakia; ^8^ Center for Medical Physics and Biomedical EngineeringMedical University of ViennaViennaAustria; ^9^ Institute of Electrodynamics, Microwave and Circuit EngineeringVienna University of TechnologyViennaAustria; ^10^ IRCCS Fondazione Ospedale San CamilloVeneziaItaly; ^11^ Center for Life Nano Science at SapienzaIstituto Italiano di TecnologiaRomeItaly

**Keywords:** Aging skeletal muscle, electrical stimulation, mitochondria Ca^2+^ uptake

## Abstract

Age‐related sarcopenia is characterized by a progressive loss of muscle mass with decline in specific force, having dramatic consequences on mobility and quality of life in seniors. The etiology of sarcopenia is multifactorial and underlying mechanisms are currently not fully elucidated. Physical exercise is known to have beneficial effects on muscle trophism and force production. Alterations of mitochondrial Ca^2+^ homeostasis regulated by mitochondrial calcium uniporter (MCU) have been recently shown to affect muscle trophism in vivo in mice. To understand the relevance of MCU‐dependent mitochondrial Ca^2+^ uptake in aging and to investigate the effect of physical exercise on MCU expression and mitochondria dynamics, we analyzed skeletal muscle biopsies from 70‐year‐old subjects 9 weeks trained with either neuromuscular electrical stimulation (ES) or leg press. Here, we demonstrate that improved muscle function and structure induced by both trainings are linked to increased protein levels of MCU. Ultrastructural analyses by electron microscopy showed remodeling of mitochondrial apparatus in ES‐trained muscles that is consistent with an adaptation to physical exercise, a response likely mediated by an increased expression of mitochondrial fusion protein OPA1. Altogether these results indicate that the ES‐dependent physiological effects on skeletal muscle size and force are associated with changes in mitochondrial‐related proteins involved in Ca^2+^ homeostasis and mitochondrial shape. These original findings in aging human skeletal muscle confirm the data obtained in mice and propose MCU and mitochondria‐related proteins as potential pharmacological targets to counteract age‐related muscle loss.

## Introduction

Age‐related sarcopenia is a syndrome characterized by a progressive loss of muscle mass and strength that greatly impacts on mobility and mortality in elderly persons (Hughes et al. 2001; Aagaard et al. 2010; Cruz‐Jentoft et al. 2010; Mitchell et al. 2012; Bijlsma et al. 2013; Miljkovic et al. 2015). Contributing factors include a severe decrease in myofiber size and number as well as decrease in the amount of motor neurons (mainly of fast type) innervating muscle fibers that is partially compensated by reinnervation of surviving slow‐type motor neurons (motor unit remodeling) (Luff 1998; Mosole et al. 2014). Reduced mobility and functional limitations during aging promote a sedentary lifestyle that generates a vicious circle further worsening muscle performance and, therefore, predisposing to an increased risk of falling, disability, and mortality (Visser and Schaap 2011).

Abnormalities of mitochondrial morphology, number, and function have been suggested to play a role in age‐related changes in muscle structure and performance (Trounce et al. 1989; Rooyackers et al. 1996; Menshikova et al. 2006; Pietrangelo et al. 2015). In the skeletal muscle, intermyofibrillar mitochondria are positioned close to the Ca^2+^ release units (CRUs), specialized intracellular junctions formed by a transverse tubule (T‐tubule) flanked by two junctional membranes of the sarcoplasmic reticulum (SR) where intracellular Ca^2+^ is stored (Rizzuto et al. 1993; Boncompagni et al. 2009). CRUs are structures deputed to excitation contraction (EC) coupling, a mechanism that allows the depolarization of the plasma membrane to be transduced into release of Ca^2+^ from the SR. As Ca^2+^ entry into the mitochondrial matrix enhances ATP production by stimulating enzymes of the TCA cycle and ATP synthase activity (Denton and McCormack 1980; Denton et al. 1988; McCormack and Denton 1988; Robb‐Gaspers et al. 1998; Rizzuto et al. 2012), the proper positioning of mitochondria adjacent to CRUs is physiologically important to rapidly sense intracellular Ca^2+^ changes that are generated during muscle contractions. Indeed, several evidences both in vitro and in vivo have demonstrated that during muscle contraction Ca^2+^ concentration in the mitochondrial matrix is increased (Brini et al. 1997; Rudolf et al. 2004; Rossi et al. 2011; Yi et al. 2011). We have shown that the number of CRUs is decreased in aging muscle (Boncompagni et al. 2006) and that the association of mitochondria with CRUs is also drastically reduced (Boncompagni et al. 2006; Pietrangelo et al. 2015).

The molecular identity of the highly selective channel responsible for Ca^2+^ entry into mitochondria, the mitochondrial calcium uniporter (MCU), was recently identified (Baughman et al. 2011; De Stefani et al. 2011) and the importance of MCU‐dependent mitochondrial Ca^2+^ accumulation in regulating skeletal muscle function was confirmed by the identification of a mutation of MICU1, one of the regulatory subunits of the MCU channel, in patients affected by proximal muscle weakness (Logan et al. 2014). In addition, overexpression or knocking down MCU in skeletal muscles of rodents was recently shown to regulate muscle growth triggering hypertrophy or atrophy, respectively (Mammucari et al. 2015).

Physical activity modulates signaling pathways involved in fiber type and muscle growth (Mammucari et al. 2007) also via intracellular Ca^2+^ (Serrano et al. 2001; McCullagh et al. 2004; Sandri et al. 2004) and that it induces specific mitochondrial adaptations. These activity‐dependent physiological effects rely on the type of exercise (i.e., aerobic endurance vs. resistance strength), as well as on its frequency, intensity, and duration (Hoppeler and Fluck 2003; Egan and Zierath 2013). Exercise training also impacts on mitochondria dynamics inducing fusion and fission phenomena to sustain cellular energy requirements (Bori et al. 2012; Iqbal et al. 2013; Konopka and Sreekumaran Nair 2013). Fusion and fission events are responsible for mitochondrial shape under the control of a core of dynamin‐related large GTPases that fuse and divide the mitochondrial membranes (Griparic and van der Bliek 2001). In particular, fission occurs upon the recruitment of dynamin‐related protein 1 (DRP1) (Cereghetti et al. 2008), while fusion is controlled by mitofusins (MFN) 1 and 2 and by optic atrophy 1 (OPA1) (Chen et al. 2003; Santel et al. 2003; Cipolat et al. 2004). OPA1 also regulates mitochondrial adaptations to bioenergetic conditions at the level of inner‐membrane ultrastructure and cristae shape. Indeed, sOPA1‐mediated effects on mitochondria respiratory efficiency is critical for muscle function as its mild overexpression prevents muscle loss after denervation (Civiletto et al. 2015; Varanita et al. 2015).

However, the ability to perform physical exercise can be limited in certain pathological conditions, therefore, alternative interventions are needed. Neuromuscular electrical stimulation (ES) was demonstrated to improve muscle mass and performance of sedentary elderly people (Bax et al. 2005; Kern et al. 2014; Mosole et al. 2014; Zampieri et al. 2014, 2015) as well as to improve muscle ultrastructure, trophism, and function in other different disorders characterized by severe muscle atrophy such as permanent upper and lower motor neuron denervation (Kern et al. 2004, 2008, 2010). Our recent data on skeletal muscle biopsies from 9 weeks trained sedentary seniors by ES in comparison to leg press (LP) showed that ES ameliorate muscle trophism, also improving muscle strength and performances (Kern et al. 2014; Zampieri et al. 2015).

In this study we investigate the impact of strength exercise protocols on MCU expression and mitochondria dynamics in a subgroup of ES‐ and LP‐trained subjects recruited for our previous studies (Kern et al. 2014; Zampieri et al. 2015). Our results show that the beneficial effects of these trainings, in particular of neuromuscular ES, rely on pathways regulating muscle mass and are associated with enhanced MCU and OPA1 protein expression. Our findings identify these mitochondrial proteins as downstream targets of physical exercise in sedentary elderlies.

## Methods

### Ethical approval and study design

Approval from the Ethical Committees of the City of Vienna for medical ethics was obtained at the study outset (EK 08‐102‐0608). The study conformed the standards set by the Declaration of Helsinki. Subjects were sedentary healthy volunteers who gave written informed consent. At enrollment they were randomly assigned to two groups and trained for 9 weeks, 2–3 times a week, with either neuromuscular ES of the anterior thigh quadriceps muscles (*n* = 10, M:F 5:5; 71.4 ± 7.1 years) or LP (*n* = 7, M:F 4:3; 70.1 ± 2.9 years).

### Training protocols and assessments

Electrical stimulation training was performed using a custom‐designed device (Krenn et al. 2011). ES‐induced muscle contraction was evoked at 60 Hz by 3.5‐sec train of impulses, separated by 4.5‐sec off intervals. Left and right thigh were stimulated in an alternative manner (Sarabon et al. 2013; Kern et al. 2014). In all subjects, ES induced a tetanic contraction of the quadriceps muscle. Additional ankle weights were also used starting from the third week of training onward as described in details (Kern et al. 2014). The intensity of the ES training was about 40% of the maximal voluntary contraction. LP was performed on a computer‐controlled LP machine using the proprioceptive vibrational mode (Kern et al. 2011). The subject was asked to push as hard as possible against the pedal. The intensity of the LP training was about 90% of maximal voluntary contraction.

Maximal isometric torque and the time which the subject needed to rise from a chair with arms folded across the chest (5× chair rise test) were measured at enrollment and at the end of the 9‐week training period as described (Sarabon et al. 2013; Kern et al. 2014).

### Muscle biopsies

Needle muscle biopsies were harvested through a small skin incision (6 mm) from the right and left vastus lateralis muscles of each subject in both groups before and after 9 weeks of training as described (Kern et al. 2014). Post‐training muscle biopsies were harvested 7 days after the last training sessions in order to analyze the long‐lasting effects of the training. Specimens collected were fixed and embedded for either light or electron microscopy (EM) as previously described (Pietrangelo et al. 2015; Zampieri et al. 2015).

### Light and quantitative histological analyses

Serial cryosections (8 *μ*m) from frozen muscle biopsies were mounted on polysine^™^ glass slides, air‐dried, and stained either with Hematoxylin and Eosin (H&E) or conventional techniques for myofibrillar ATPase (mATPase) to evaluate tissue morphology and muscle fiber type. Morphometric analyses to calculate the minimum transverse myofiber diameter were performed on stained cryosections using Scion Image for Windows version Beta 4.0.2 (2000; Scion Corporation, U.K.) as described (Rossini et al. 2002; Kern et al. 2010, 2014) and the results were reported as mean ± SD in pre‐ and post‐training group of muscle biopsies. To give an expression of the number of very small fibers, the atrophy factor was calculated in pre‐ and post‐training muscle biopsies (Table 2) as described by Dubowitz (1985). Briefly, to put the results in a proportional basis, the total number of fibers having diameter between 40 and 30 *μ*m, 30 and 20 *μ*m, 20 and 10 *μ*m, and less than 10 *μ*m was counted and multiplied by one, two, three, and four, respectively. The products were then added together and normalized to the total number of analyzed myofibers.

### Immunofluorescence staining

Serial cryosections (8 *μ*m) from frozen muscle biopsies were also labeled for either fast or slow myosin heavy chain (MHC) (product numbers – NCL‐MHCs and NCL‐MHCf; 1:10; Novocastra, Newcastle upon Tyne, U.K.) and laminin (product number L9393, 1:100; Sigma‐Aldrich, St. Louis, MO) as described (Kern et al. 2014; Mosole et al. 2014). Secondary anti‐rabbit or anti‐mouse Alexa 488 or 594 antibodies (product numbers A11001, A11005, 1:200; Life Technologies, Carlsbad, CA) and anti‐rabbit FITC conjugated antibody (product number F1262, 1:200; Sigma‐Aldrich) were used. Coverslips were mounted onto the glass slides using ProLong Gold antifade reagent with DAPI to counterstain nuclei (Life Technologies) and observed under the fluorescent microscope.

### Ultrastructural quantitative analyses of mitochondria by EM

Ultrathin sections (50 nm) were cut from muscle biopsy samples embedded for EM with a Leica Ultracut R (Leica Microsystem, Vienna, Austria) using a Diatome diamond knife (Diatome Ltd., Biel, Switzerland). Sections were then stained in 4% uranyl acetate and lead citrate solutions. Sections were examined with a FP 505 Morgagni Series 268D electron microscope (FEI Company, Brno, Czech Republic) at 60 kV, equipped with a Megaview III digital camera and AnalySIS software (Olympus Soft Imaging Solutions GmbH, Munster, Germany). In each specimen six fibers were analyzed. In each fiber six micrographs of nonoverlapping regions were randomly collected from longitudinal sections at 14,000× of magnification for the following quantificative analyses: (1) the relative fiber volume occupied by mitochondria was determined using the well‐established stereology point technique (Loud et al. 1965; Mobley and Eisenberg 1975). Briefly, after superimposing an orthogonal array of dots at a spacing of 0.20 *μ*m to the electron micrographs, the ratio between numbers of dots falling within mitochondrial profiles and total number of dots covering the whole image was used to calculate the relative fiber volume occupied by mitochondria (Table 3, column a). (2) Mitochondrial density was evaluated from electron micrographs of nonoverlapping regions randomly collected from longitudinal sections and reported as average number over 100 *μ*m^2^ (Table 3, column b). In each EM micrographs, we also determined mitochondrial positioning with respect to the I and A bands: if an individual mitochondrion extended from one I band to another, it was counted in both bands (Table 3, column c). (3) Mitochondrial average size was also evaluated (Table 3, column d) using AnalySIS software (Olympus Soft Imaging Solutions GmbH) by manually tracing only clearly discernible outlines of mitochondria.

### Immunoblotting

Protein lysates were prepared from 10 cryosections (20 *μ*m thick) by means of Qiagen Tissue Lyser (Qiagen GmbH, Hilden, Germany) in a buffer containing 50 mmol/L Tris pH 7.5, 150 mmol/L NaCl, 5 mmol/L MgCl2, 1 mmol/L DTT, 10% glycerol, 2% SDS, 1% Triton X‐100, Roche Complete Protease Inhibitor Cocktail (Roche Diagnostics S.p.a., Monza, MB, Italy), 1 mmol/L PMSF, 1 mmol/L NaVO_3_, 5 mmol/L NaF, and 3 mmol/L *β*‐glycerophosphate. Protein concentration was determined by the colorimetric detection of the cuprous cation (Cu^1+^) by bicinchoninic acid method (Pierce^™^ BCA assay; Thermo Scientific, Rockford, IL), subsequently separated on 4–12% gradient SDS‐PAGE and electrotransfered onto nitrocellulose membrane which was then probed with different antibodies: MCU (product number HPA016480, 1:500; Sigma‐Aldrich), Actin (product number A2066, 1:15000; Sigma‐Aldrich); TOM20 (product number sc11415; 1:1000; Santa Cruz Biotechnology, Segrate, MI, Italy); SDH‐A (product number #11998, 1:500; Cell Signaling Technology; Euroclone, Pero, MI, Italy), COX IV (product number #4844, 1:1000; Cell Signaling Technology), OPA1 (product number 612606, 1:2000; BD Biosciences, Milano, Italy), and Mitofusin 2 (product number ab50838, 1:1000; AbCam, Cambridge, U.K.). Signals were visualized via chemiluminescence as described (Zampieri et al. 2001).

### Gene expression analyses

Total RNA was extracted from muscle using tissue lyser (Qiagen GmbH) in TriReagentTM (Sigma‐Aldrich) and it was reverse transcribed using a QuantiTect Reverse Transcription Kit (Qiagen GmbH). Quantitative RT‐PCR was performed on an ABI PRISM 7500 SDS (Applied Biosystems, Foster City, CA) using premade 6‐carboxyfluorescein (FAM)‐labeled TaqMan assays for GAPDH, IGF‐1 Ea, IGF‐1 Eb, IGF‐1 Ec, IGF‐1 pan (Applied Biosystems), and for Atrogin1, MuRF1, PGC1a, and PGC1a4, MCU, DRP1, Mitofusin1 and 2, and OPA1 as described (Brocca et al. 2012; Varanita et al. 2015). Quantitative sample values were normalized to the expression of GAPDH mRNA. Relative levels for each gene were calculated using the 2‐DDCt method (Livak and Schmittgen 2001) and reported as mean fold change in gene expression.

### Statistical analyses

The statistical significance of data collected by EM analyses was determined using a Student's *t* test (Microcal Origin^®^ 6.0; Microcal Software, Inc., Northampton, MA), while statistical significance of percentage values was investigated using a chi‐squared test (Microsoft^®^ Office Excel^®^ 2007; Microsoft Corporation). Statistical analysis of morphometric, densitometric, and gene expression datasets was performed with GraphPad Prism v5.0 software (GraphPad Software, Inc., La Jolla, CA); statistical significance of average numbers was determined using Wilcoxon matched pairs test. Values of *P* < 0.05 were considered significant.

## Results

### Skeletal muscle biopsies from sedentary seniors before training show specific features of aging

**Figure 1:**
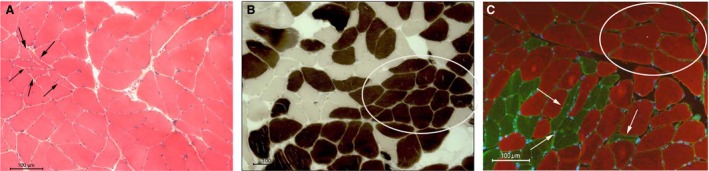
Specific features of aging can be observed in muscle biopsies before the training. (A) H&E staining. Black arrows point at small angular and flat‐shaped myofibers. (B) mATPase histochemistry for slow‐type fibers (black). Slow fiber‐type grouping is encircled. (C) Antifast (green) and slow (red) myosin heavy‐chain coimmunofluorescence. Sarcolemma is stained in green with antilaminin antibody, whereas nuclei are counterstained in blue with DAPI. White arrows point at severely atrophic fast‐type fibers. Slow fiber‐type grouping is encircled. In all images scale bar = 100 *μ*m.

Muscle biopsies of 70‐year‐old sedentary seniors were collected before and after the training and morphological, biochemical, and molecular analyses were performed. Histological analyses of pretrained muscles showed some severely atrophic, flat shaped, and angulated fibers (arrowed in panel A, Fig. 1), presenting typical signs of denervation. Histochemical analyses testing for mATPase activity revealed the presence of slow twitching myofibers (dark stained in panel B, Fig. 1) organized in cluster (type grouping, i.e., one myofiber completely surrounded by fibers of the same phenotype, white encircled in panels B, Fig. 1), and constituted by medium or large fibers. Immunofluorescence analyses for fast and slow MHC demonstrated that fast fibers (green stained in panel C, Fig. 1) were smaller than slow ones (red stained in panel C, Fig. 1). Moreover, fast atrophic fibers displayed an angular or flat shape features (white arrowed in panel C, Fig. 1). These morphological aspects were almost absent in slow‐type fibers (red stained in panel C, Fig. 1).

### Neuromuscular ES training significantly increases maximal isometric torque and strength of sedentary elderlies without damaging their muscles

Several functional and mobility tests can be used to assess frailty, and degree of independence in elderlies (Mosole et al. 2014; Zampieri et al. 2014, 2015). Among these, maximal isometric torque and chair rise are specific tests for muscle strength, which represents a key factor for fall prevention in aging (Bohannon 1997; Cruz‐Jentoft 2013).

**Figure 2:**
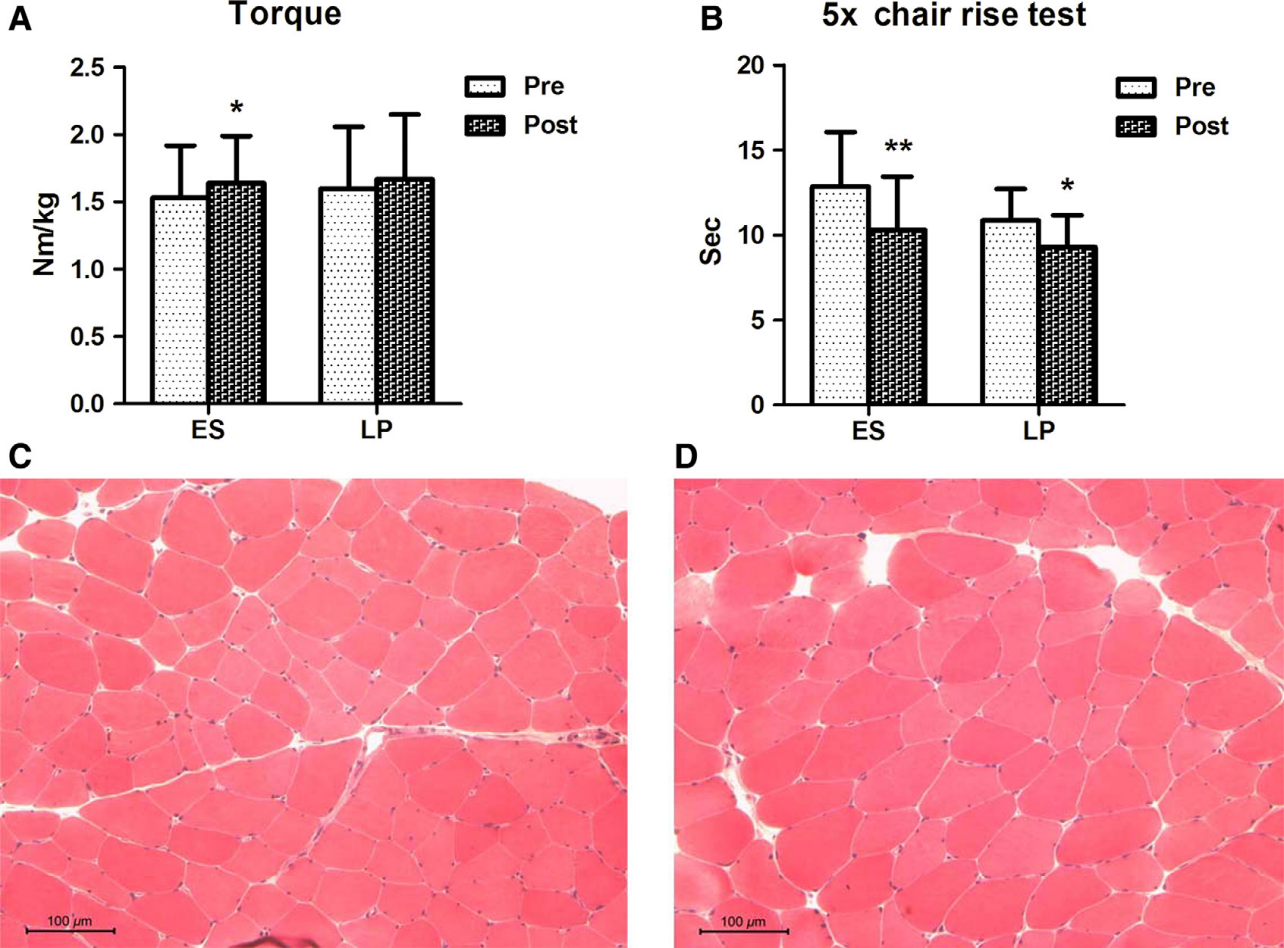
Functional and morphological changes in aging muscles after 9 weeks of physical exercise. (A) Muscle torque (*P = 0.026) and (B) 5× chair rise tests (**P = 0.036, *P = 0.050) in pre‐ and post‐training conditions (ES,* n* = 10; LP,* n* = 7). Values are given as mean ± SD. Absence of necrosis, inflammation, or increased number of centrally nucleated myofibers in electrical stimulation (ES) (C) and leg press (LP) (D) trained muscle. H&E staining, scale bar = 100 *μ*m.

In line with our previous results (Kern et al. 2014; Zampieri et al. 2015), both groups of trained subjects improved their functional tests performances (Fig. 2, panel A). Torque values increased and 5× chair rise test's score was decreased when compared to the pretraining conditions as an indication of improved muscle strength. These changes were more pronounced and statistically significant in subjects trained with neuromuscular ES (Fig. 2, panel A). Inflammation, bands of hypercontraction, centrally nucleated fibers, or fibrosis was not detected in post‐training muscle biopsies (Fig. 2, panel B), suggesting that these exercise protocols did not injury aging weak muscles.

The overall training load was calculated in both groups of subjects and expressed as the net muscle contraction time (MCT) over the 9 weeks of training as reported in Table 1. In ES‐trained group, the total MCT was higher in comparison to LP (144 vs. 48 min), while the intensity of training was much higher in LP with respect to ES (90% vs. 40% of the maximal voluntary contraction, respectively).

**Table 1:** Muscle contraction time in ES and LP training

	Weeks of training (*n*)	Sessions/week (*n*)	MCT/session (min)	MCT/week (min)
ES	3	2	6	36
	6	3	6	108
			Total MCT	**144**
LP	3	2	2	12
	6	3	2	36
			Total MCT	**48**

ES, electrical stimulation; LP, leg press; MCT, muscle contraction time on the LP was calculated multiplying the net time for one repetition by the number of repetitions performed in each session. In ES training, MCT was the time while ES‐evoked muscle contraction‐induced knee extension with an angle less than 30°. In bold are highlighted the total MCT for each condition (ES vs. LP).

### Neuromuscular ES maintains myofiber size, inducing a recovery of severely atrophic fast‐type fibers

**Figure 3:**
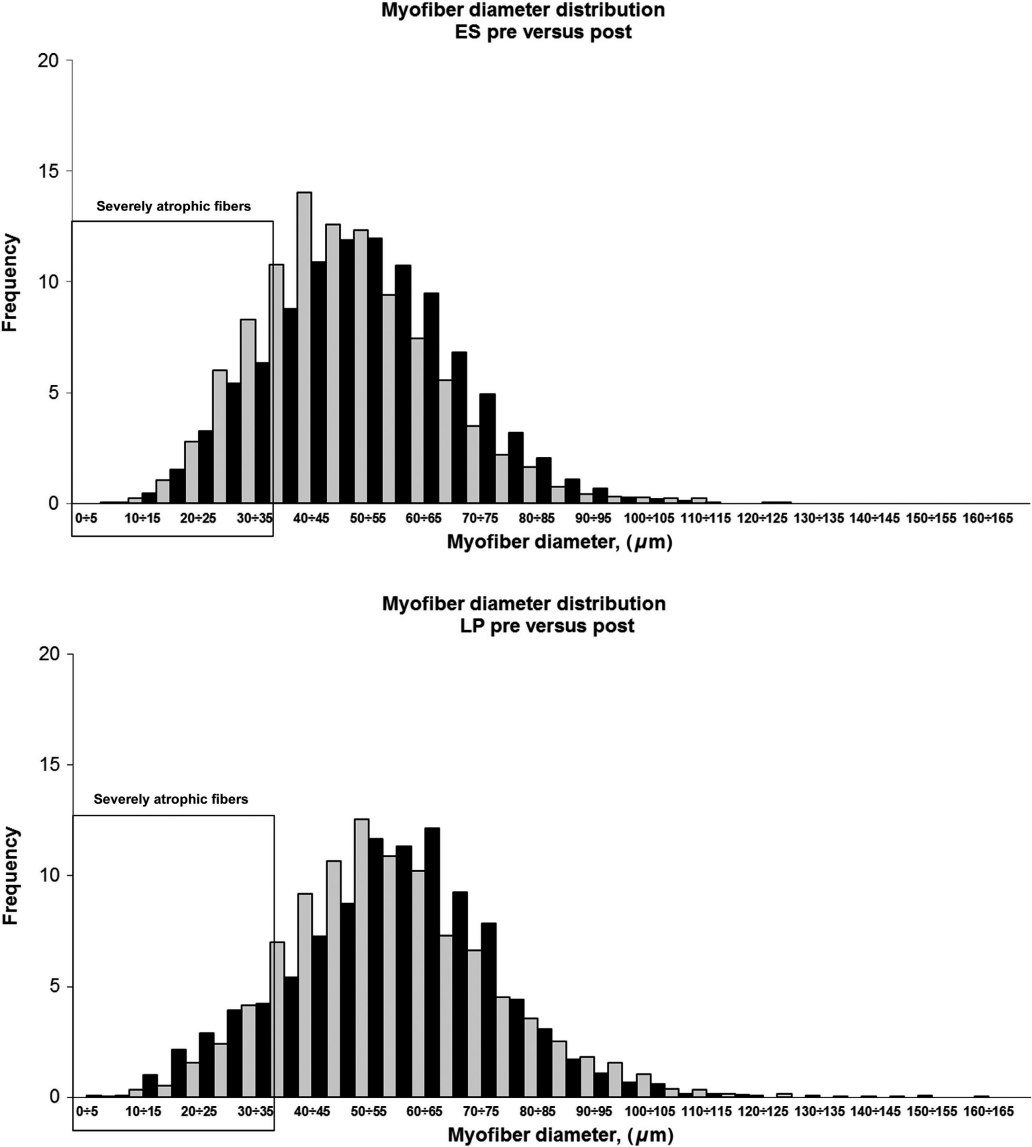
Morphometrical analyses in skeletal muscle biopsies before and after electrical stimulation (ES) and leg press (LP) trainings. Myofiber diameter distribution in ES (A, *n* = 10) and LP (B, *n* = 7) muscle biopsies pre‐ (white bars) and post‐ (black bars) training. The frequency of fibers having diameter ranging between 0 and 165 *μ*m is reported grouped by 5 *μ*m diameter.

In ES group the average myofiber size slightly increased (49.16 ± 15.80 vs. 51.01 ± 16.38; *P* < 0.0001) while atrophy factor decreased after the training (Table 2, 404 vs. 384, pre vs. post), indicating that ES protocol was effective in the recovery of severely atrophic fibers. The major trophic effect was observed on those fibers having diameter between 25 and 45 *μ*m as shown by the spectrum of myofiber size distribution: the frequency of the fibers in this range decreased in post‐training muscle biopsies (Fig. 3, panel A, black bars) in comparison to the pre‐training ones (Fig. 3, panel A, white bar), while the frequency of those having diameter >55 *μ*m increased after the training. Interestingly, the recovery was predominantly observed in fast‐type population (Table 2, 585 vs. 412, pre vs. post). In LP group, the average myofiber size significantly decreased after the training (57.87 ± 19.17 vs. 55.21 ± 18.13, *P* < 0.0001) and consistently atrophy factor increased (Table 2, 233 vs. 333, pre vs. post). A minor effect of LP training on the rescue of fast‐type myofiber atrophy was observed (Table 2, 395 vs. 379, pre vs. post). These results indicate that neuromuscular ES was more efficient to promote muscle hypertrophy and that fast‐type fibers were more responsive to ES‐ and LP‐induced physical activity in aging muscles.

**Table 2:** Atrophy factor in pre‐ and post‐training muscle biopsies. Calculation of atrophy factor (as described in Material and Methods section) in pre‐ and post‐training muscle biopsies revealed that ES physical exercise had major effects on the recovery of severely atrophic fibers, in particular of fast type, while LP had milder trophic effects, despite the observed improvements in muscle torque and strength

	Pretraining		Post‐training		Rescue of AF
	AF	Total fibers	AF	Total fibers	Δ %
ES trained					
All fibers	404	3286	384	5023	+5
Fast type	585	1765	412	3294	+42
Slow type	269	1575	394	2664	−32
LP trained					
All fibers	233	2367	333	2525	−30
Fast type	395	1074	379	1266	+4
Slow type	192	1570	311	1822	−38

AF, atrophy factor; ES, electrical stimulation; LP, leg press.

### Neuromuscular ES triggers the induction of IGF1 and a concomitant suppression of atrophy‐related genes

**Figure 4:**
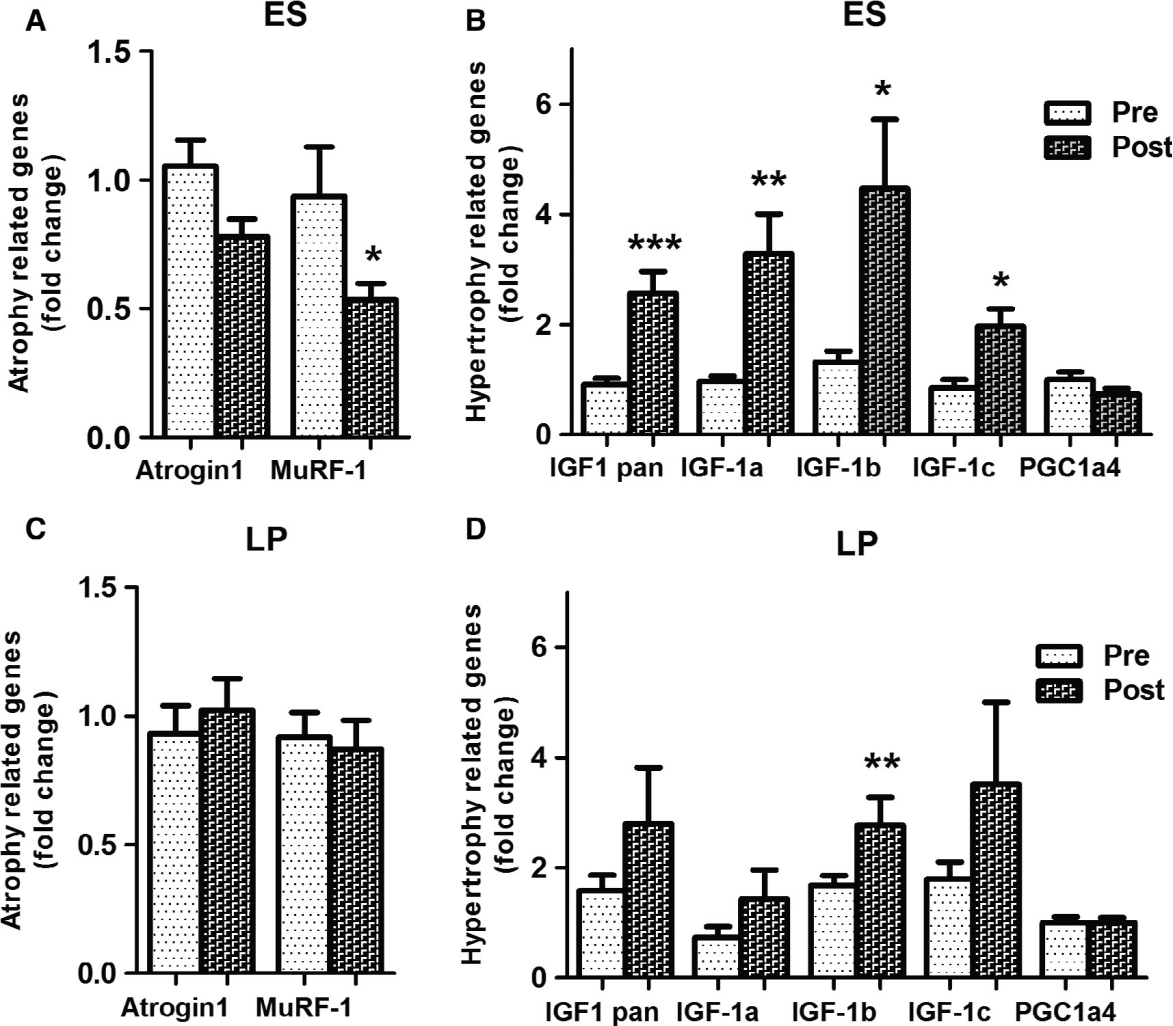
Electrical stimulation (ES)‐mediated morphological and functional improvements are sustained by a significant upregulation of hypertrophy‐related genes and downregulation of atrophy‐related ones. Gene expression analyses in muscle biopsies pre‐ versus post‐ES (A–B, *n* = 10) and LP (C–D, *n* = 7) trainings. Data are reported as mean ± SD (panel A *P = 0.031, panel B IGF1 pan ***P = 0.001, IGF‐1a** *P* = 0.001, IGF‐1b *P = 0.014, IGF‐1c *P = 0.013; panel D **P = 0.002).

Muscle size depends on mechanical stimulation and the mechanical load modulates anabolic and catabolic signaling pathways. In order to investigate the molecular mechanisms underlying the morphological and functional changes, we monitored gene expression of the atrophy‐related genes Atrogin1 and MuRF1 and of the growth‐promoting hormone insulin growth factor‐1 (IGF1) and its isoforms. The expression of the new splicing variant transcript from PGC‐1a gene, the isoform 4 (PGC1a4), that has been recently reported to trigger muscle hypertrophy (Ruas et al. 2012; Mammucari et al. 2015) was also measured. In line with our previous study (Kern et al. 2014) in ES‐trained group we observed a significant downregulation of MuRF1 and only a trend of Atrogin1 reduction, while simultaneously all the IGF1 isoforms were significantly upregulated (Fig. 4, panel A). These molecular changes suggest that ES activated a program of gene expression that counteracts muscle atrophy and promotes muscle growth. Conversely, LP training did not affect the levels of Atrogin1 and MuRF1, while it significantly induced, even if to a less extend, the expression of IGF1b isoform (Fig. 4, panel B). These results are in agreement with the minor effects on function and myofiber size that were observed in LP‐trained subjects (Figs. 2 and 3). In both groups, PGC1a4 was unaffected by the training, suggesting that the effect on myofiber size observed after 9 weeks of training mainly relies on IGF1‐signaling pathway (Fig. 4, panels A and B).

### Physical exercise induces an increase in MCU protein content

**Figure 5:**
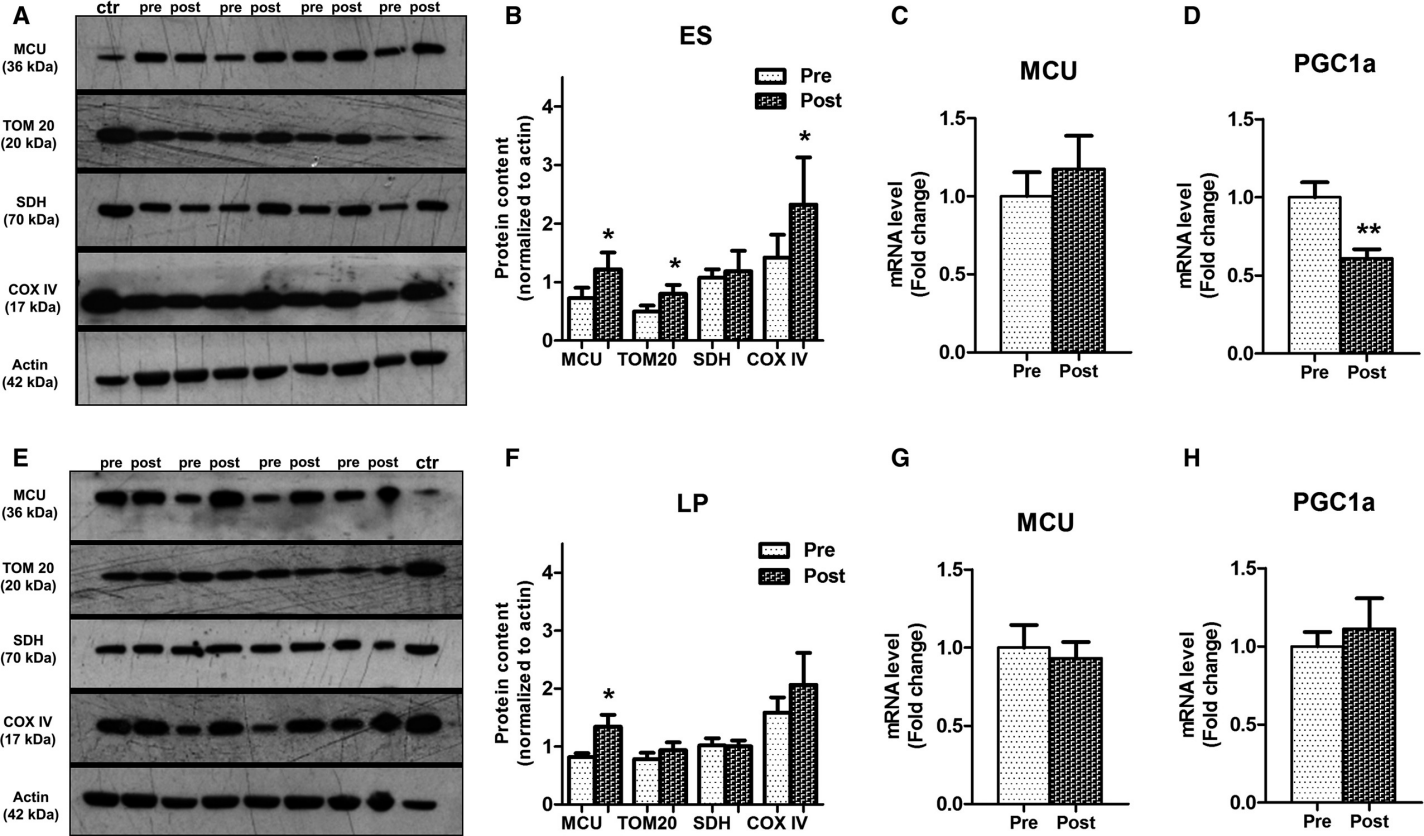
Nine weeks of physical exercise induced a significant increase in mitochondrial calcium uniporter (MCU) protein content, associated with increased COX IV expression level in electrical stimulation (ES)‐trained group. Representative immunoblots for MCU, TOM20, and respiratory chain enzymes SDH and COXIV on muscle homogenates from pre‐ and post‐ES (A) (*n* = 10) and leg press (LP) (E) (*n* = 7) training conditions. Ctr = homogenates from mouse tibialis anterior muscle overexpressing MCU. Densitometric quantification of detected proteins in all analyzed muscle homogenates from ES (B) and LP (F) samples, normalized to actin. Values are given as mean ± SD; Exact mean, SD, and *P* values are reported in Table 3. mRNA expression levels of MCU (C and G) and PGC1a (D and H) as a marker of mitochondrial biogenesis from ES (C–D) (*n* = 10) and LP (D–H) (*n* = 7) pre‐ and post‐training muscle biopsies. Values are given as mean ± SD; (panel D **P = 0.0020).

Exercise‐dependent muscle activity induces intracellular Ca^2+^ release from the CRUs. This calcium is uptaken by the intermyofibrillar mitochondria where it affects the respiratory chain enzymes to sustain the energy demand of contraction. Western blot analyses on muscle homogenates from biopsies collected before and after 9 weeks of training revealed a significant increase in MCU protein content in response to exercise (Fig. 5, panels A–B and E–F, Table 3), with no significant changes in transcript level (Fig. 5, panels C and G) indicating a post‐transcriptional regulation of this protein. COX IV respiratory chain enzyme significantly increased only in ES‐trained group (Fig. 5, panels A and B, Table 3), whereas SDH protein levels were unchanged in both post‐training conditions (Fig. 5, panels A–B, and E–F, Table 3). Importantly, the significant increase in MCU and COX IV protein expression levels was observed in the 80% of the subjects (Table 3) indicating that these changes are a generalized effect of the training in the great majority of the subjects. TOM20 protein, which was used as marker of outer mitochondrial membrane and to monitor mitochondrial mass, significantly increased in ES‐trained subjects (Fig. 5, panels A and B, Table 3).

**Table 3:** MCU and other mitochondrial protein expression levels in skeletal muscle biopsies before and after ES and LP training. Quantitative analyses of Western blot on muscle homogenates revealed that 9 weeks of physical exercise significantly increased MCU protein expression levels. ES training induced also a significant increase in COX IV respiratory chain enzyme and mitochondrial fusion protein OPA1. The great majority of the subjects experienced the reported mean changes, indicating that the induction of protein expression levels is a generalized response to the training

	Pre (mean ± SD)	Post (mean ± SD)	*t* test	Subjects showing the indicated changes (%)
ES				
MCU	0.68 ± 0.52	1.13 ± 0.86	0.027	80
SDH	1.07 ± 0.46	1.19 ± 1.10	n.s.	50
COX IV	1.42 ± 1.22	2.33 ± 2.55	0.049	80
TOM20	0.49 ± 0.28	0.80 ± 0.45	0.048	80
OPA1	0.73 ± 0.47	1.08 ± 0.41	0.040	86
Mtf2	0.80 ± 0.28	0.78 ± 0.43	n.s.	57
LP				
MCU	0.82 ± 0.17	1.34 ± 0.55	0.020	100
SDH	1.02 ± 0.33	1.00 ± 0.28	n.s.	43
COX IV	1.59 ± 0.69	2.07 ± 1.46	n.s.	43
TOM20	0.74 ± 0.21	1.02 ± 0.37	n.s.	71
OPA1	1.05 ± 0.56	0.78 ± 0.49	0.047	86
Mtf2	0.75 ± 0.25	0.67 ± 0.23	n.s.	71

Values are shown as mean ± SD. ES, electrical stimulation; LP, leg press; MCU, mitochondrial calcium uniporter.

However, PGC1a was significantly downregulated after ES (Fig. 5, panel D) while was unchanged after LP training (Fig. 5, panel H).

### Neuromuscular ES or LP trainings differently impact on mitochondria network dynamics

**Figure 6:**
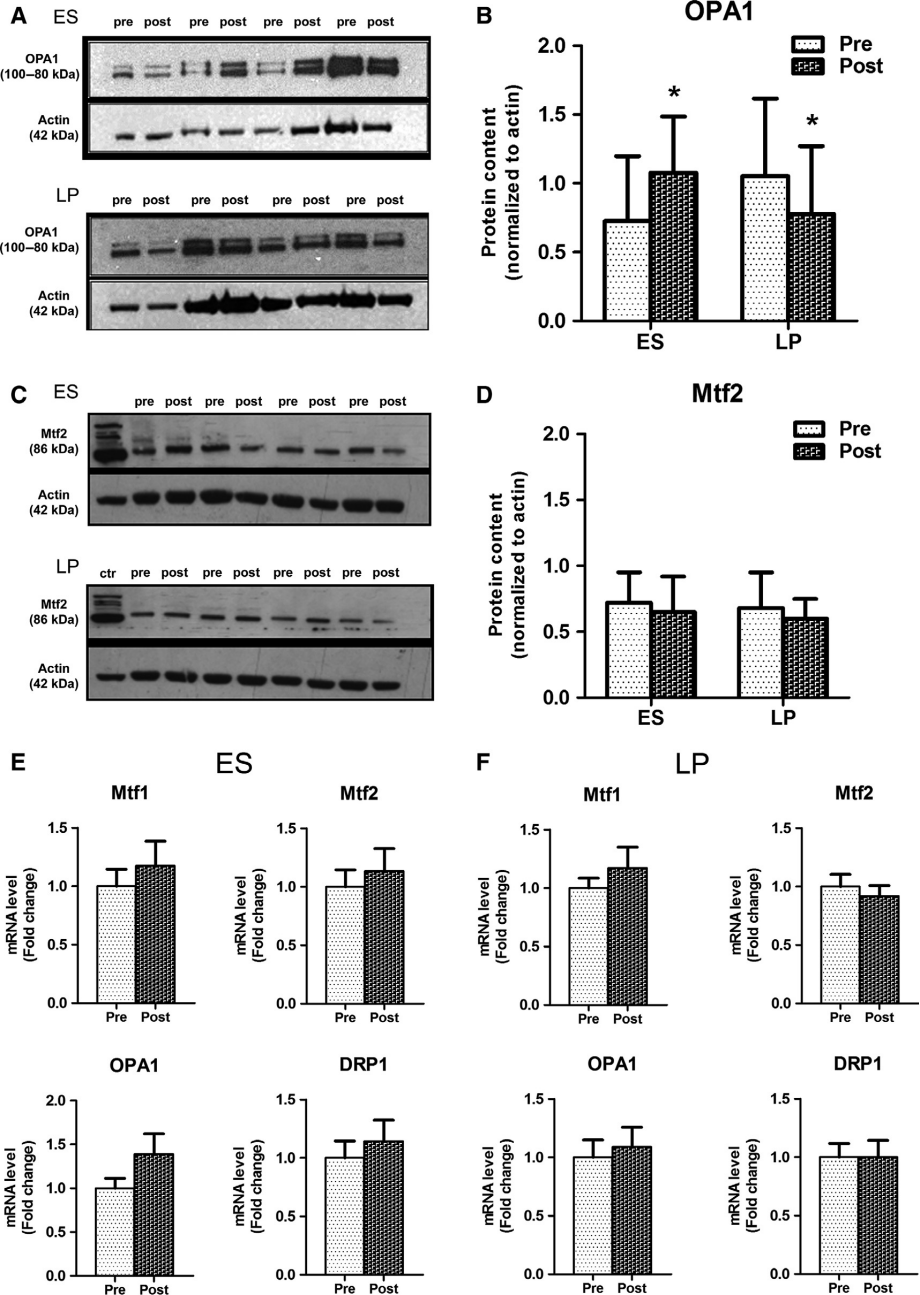
Electrical stimulation (ES) training induced a significant increase in OPA1 mitochondrial fusion protein. Representative immunoblot analyses for OPA1 (A) and Mtf2 (C) in pre‐ and post‐training conditions (*n* = 7). Ctr = HeLa total cell lysate. Densitometric quantification of Western blot from muscle homogenates of all analyzed samples showing OPA1 (B) and Mtf2 (D) protein expression levels in pre‐ and post‐training conditions, normalized to actin. Values are given as mean ± SD. Exact mean, SD, and *P* values are reported in Table 3. (F) Fold changes in genes regulating mitochondria dynamics after ES (E) and leg press (LP) (F) training. Values are given as mean ± SD;* P* = no significant changes for all analyzed genes.

Qualitative observation by EM did not reveal striking structural differences in biopsies from subjects trained with ES or LP between pre‐ and post‐training. However, a quantitative analyses of the mitochondrial network did reveal some morphological changes in the biopsies from the ES group between pre‐ and post‐training (Table 4). Indeed, while the relative volume occupied by mitochondria did not change following either ES and LP training (Table 4, column a), in the ES group the number of mitochondria was decreased (Table 4, column b: 48.3 ± 1.3 vs. 38.6 ± 1.2, respectively, in pre‐ and post‐training muscle biopsies; *P* < 0.0001), whereas their size was increased (Table 4, column d: 72.3 ± 1.9 vs. 80.4 ± 2.5, respectively, in pre‐ and post‐training muscle biopsies; *P* = 0.009). The statistically significant changes reported in Table 4 (columns b and d) suggest a remodeling of the mitochondrial apparatus induced specifically by the ES training, but not by the LP exercise protocol. Indeed, data of Table 4 (columns b and d) suggest that ES stimulates fusion of mitochondria into larger organelles. In order to support this interpretation, we measured the expression levels of protein‐regulating mitochondrial shape like Mtf2 and OPA1. A significant increase in OPA1 in the muscles of more than 85% of the ES‐trained subjects was detected (Table 3), while it significantly decreased in more than 85% of the subjects after LP training (Fig. 6, panels A and B, Table 3). On the other hand, Mtf2 expression was unchanged by the training (Fig. 6, panels C and D, Table 3) and also the transcript levels of Mtf1 and 2, OPA1, and DRP1 genes were unaffected after 9 weeks of training (Fig. 6E and F).

**Table 4:** EM ultrastructural analyses of intermyofibrillar mitochondria before and after ES and LP trainings. Quantitative analyses of the mitochondrial population by electron microscopy revealed that, following the ES protocol, mitochondrial number (column b) and size (column d) changes significantly (a *P* < 0.01). These changes suggest a remodeling of the mitochondrial apparatus induced specifically by the ES training, but not by the LP protocol

	(a) Mitochondria volume/total volume (%)		(b) No. of mitochondria/100 *μ*m^2^		(c) No. of mitochondria at A band/100 *μ*m^2^ (%)		(d) Mitochondrial average size (nm^2^ × 10^3^)	
	Pre	Post	Pre	Post	Pre	Post	Pre	Post
ES	3.4 ± 0.1	3.5 ± 0.1	48.3 ± 1.3	38.6 ± 1.2^a^	7.5 ± 0.5 (16)	6.7 ± 0.4 (19)	72.3 ± 1.9	80.4 ± 2.5^a^
LP	3.5 ± 0.1	3.5 ± 0.1	42.4 ± 1.5	45.7 ± 1.5	5.4 ± 0.4 (13)	5.5 ± 0.4 (13)	74.2 ± 1.8	73.4 ± 2.0

Values are shown as mean ± SEM. Sample size: 48 fibers from ES and 36 fibers from LP; 6 micrographs/fiber. EM, electron microscopy; ES, electrical stimulation; LP, leg press.

a *P* < 0.01 versus Pre.

## Discussion

The etiology of sarcopenia is multifactorial and involves several intrinsic and extrinsic factors, but the precise underlying mechanisms are poorly understood. Numerous studies have clearly shown that old age is characterized by a fiber‐type shift toward slow phenotype (Aagaard et al. 2010; Ohlendieck 2011) that can only partially account for the weakness that characterizes aging muscle (Mitchell et al. 2012; Bijlsma et al. 2013). Several histological analyses of sarcopenic muscles have shown a progressive increase in denervated myofibers, primarily of fast type, indicating that denervation is one of the important mechanisms that contribute to muscle atrophy and weakness in aging (Edström et al. 2007; Aagaard et al. 2010; Gonzalez‐Freire et al. 2014). Dysfunction of the EC coupling has also been proposed to contribute to the loss of specific force of aging muscle (Boncompagni et al. 2006). Importantly, exercise seems to counteracts these features (Mosole et al. 2014; Zampieri et al. 2014), but the mechanistic insights triggered by physical activity remain unknown. Numerous studies investigating the role of ES‐induced physical activity in muscle recovery demonstrated the beneficial effects of this strategy as a good alternative approach to voluntary exercise or traditional rehabilitation therapy (Quittan et al. 2001; Levine et al. 2013; Maddocks et al. 2013; Kern et al. 2014).

In this study the muscle biopsies collected before the training showed histological features typical of aging, such as a predominance of slow fibers, which were clustered in grouping, and several atrophic, angulated, and flat‐shaped fibers suggesting that denervation and reinnervation were present. However, 9 weeks of neuromuscular ES or LP recovered myofiber atrophy with a specific hypertrophic effect on fast‐type fibers that was predominantly observed in ES‐trained group of subjects. The morphological changes resulted in an improvement of muscle torque and strength. Nevertheless, some differences have been observed in response to the two types of trainings. ES protocol was more efficient to maintain myofiber size, counteracting atrophy and promoting muscle growth when compared to LP. This difference might be due to the two training approaches that diverge in terms of time and intensity of exercise. In fact, neuromuscular ES was applied to knee extensor muscles with a stimulation pattern designed for the submaximal activation (Sarabon et al. 2013) and a total muscle contraction time of 144 min over the 9‐week period. On the other side, LP exercised different muscles at the same time, that is, hip extensor, knee flexor, and extensor muscles. Therefore, LP intensity is higher than ES, but the time spent in training is much less with a total MCT of only 48 min. This protocol of short periods of high‐intensity contractions was specifically designed to avoid potential muscle and joint injuries. Therefore, the observed differences in terms of hypertrophy, force, and signaling pathways might be consequent to the short overall muscle contraction time of LP protocol. However, both trainings improved muscle function and, to a certain extent, also the size of fast fibers suggesting that some molecular mechanisms were shared between these two types of exercise. The pathways found to be commonly activated by these trainings are related to IGF1 and mitochondrial calcium homeostasis. In fact, we found that both ES and LP triggered IGF1 and MCU expression. However, we observed a significant increase in MCU protein content in response to exercise, with no significant changes at mRNA level, suggesting that the protein is regulated post‐trascriptionally, even though little is known about this issue at the moment. Tyrosine phosphorylation of MCU has been described to control MCU activity (O‐Uchi et al. 2014). In addition, two Ca^2+^/calmodulin‐dependent protein kinase II (CaMKII) target sites were identified in the MCU sequence (Joiner et al. 2012). However, the role of CAMKII in regulating MCU activity has been questioned (Fieni et al. 2014) and deserves deeper investigation.

Of note, ES but not LP promoted the upregulation of IGF‐1Ec isoform, which is normally upregulated in response to mechanical signals (Matheny et al. 2010). Moreover, increased levels of IGF‐1 were associated with reduced level of expression of MuRF1, a gene involved in muscle atrophy. This suggests that ES mimics physical exercise, improving molecular adaptations of muscle, counteracting muscle atrophy, and improving functional outcomes.

Interestingly, mice lacking MCU exhibit functional abnormalities in conditions that require a rapid increase in the skeletal muscle work load. In particular, a significant impairment of the exercise capacity, strength, and power output has been shown in MCU^−/−^ mice by inclined treadmill test, forearm grip strength assessment, and vertical pull up, without any apparent alterations in skeletal muscle fiber‐type composition (Pan et al. 2014). In agreement with data observed in MCU^−/−^ mice, in our ES‐ and LP‐trained subjects MCU induction was observed together with improvements in muscle torque and strength, further sustaining the role of MCU in regulating skeletal muscle work.

Indeed, the correlation among muscle contraction, MCU induction, and changes in myofiber size is of particular interest. The human data shown here are in good agreement with the recent findings about MCU involvement in muscle mass regulation, in rodents. Indeed, we have recently found that overexpression of MCU in adult muscle promotes hypertrophy while knocking down MCU triggers muscle atrophy (Mammucari et al. 2015). Therefore, the induction of MCU might contribute to muscle growth via a mitochondria‐ and energy‐dependent signaling.

Electron microscopy ultrastructural analyses showed that mitochondria volume when normalized to myofiber volume was unchanged after ES and LP training. However, following the ES (but not LP) training the number of mitochondria was decreased while their size became bigger, suggesting fusion of mitochondria into larger organelles.

The observed ultrastructural changes in mitochondrial network suggest an involvement of mitochondrial shaping machinery and indeed we found an increase in OPA1 protein but not of Mfn2. Several functional differences have been reported between Mfn1/Mfn2 and OPA1 in terms of mitochondrial fusion, localization, bioenergetics, and shape (Cipolat et al. 2004). Mfn2 controls outer mitochondrial membrane fusion, but also tethers of mitochondria to the endoplasmic reticulum (de Brito and Scorrano 2008), while OPA1 regulates inner mitochondrial membrane fusion as well as cristae shape and supercomplexes assembly. Importantly, mild OPA1 upregulation elicits several beneficial effects in terms of tissue physiology. In fact, we have recently found that expression of OPA1 is sufficient to counteract muscle loss after denervation (Varanita et al. 2015) but does not induce muscle hypertrophy in basal condition. Altogether, these findings suggest that OPA1 is involved in metabolic/bioenergetic changes that are important for muscle maintenance and regulation in stress conditions. The OPA1‐dependent beneficial effects might be dissociated from its profusion activity as PGC1a was downregulated and Mfn2 did not change after ES training.

Altogether, our results indicate that while both exercise protocols ameliorated some functional parameters and increased MCU expression, only ES induced OPA1 expression, changes in mitochondrial network, and big improvements in fiber size and muscle strength. Therefore, we can speculate that the increase in MCU expression induced by physical activity is associated with hypertrophic signaling, while the changes in mitochondria dynamics are synergistic with MCU and linked to metabolic adaptations and energy production. LP training protocol was probably too mild and/or too short in overall time period to have significant impact on muscle morphometry and mitochondria dynamics in aged muscles.

In conclusion, our findings show for the first time that MCU and OPA1 expressions are modulated by physical exercise in aging human muscles and, therefore, suggest that mitochondria can serve as the sensors and retrogradely induce nuclear programs to regulate muscle mass.

Further experiments are needed to dissect the mechanistic insights that connect exercise to mitochondria and to gene/protein expression. Understanding this link will allow‐the development of novel therapeutic strategies to counteract sarcopenia and to promote healthy aging.

## Conflict of Interest

All authors declare no conflict of interest.
