# Erratum

**DOI:** 10.14814/phy2.13229

**Published:** 2017-03-29

doi: 10.14814/phy2.13005

In (Zampieri et al. 2016), the following error was published in the author affiliations section.

The author affiliation of Rosario Rizzuto mentioned in the final version is:

2 Venetian Institute of Molecular Medicine, Padova, Italy.

This is incorrect and should instead be:

3 Department of Biomedical Sciences, University of Padova, Padova, Italy.

We apologize for the error.
